# A Resiliency Intervention to Support Nurses Engaged in the Provision of HIV Care in KwaZulu-Natal, South Africa: Protocol for a Pilot Randomized Controlled Trial

**DOI:** 10.2196/79777

**Published:** 2026-06-25

**Authors:** Christina Psaros, Jennifer A Smit, Nzwakie Mosery, Lara N Traeger, Sanelisiwe Mngomezulu, Bongeka Qiya, Busi Maphumulo, Amelia M Stanton, Zoya-Maria R Aoun, C Andres Bedoya, Elyse R Park

**Affiliations:** 1 Department of Psychiatry, Massachusetts General Hospital, One Bowdoin Square, 9th Floor, Boston, MA, 02114, United States, 1 617 726 7458; 2 Harvard Medical School, Boston, MA, United States; 3 Department of Obstetrics and Gynaecology, MatCH Research Unit (MRU), University of the Witwatersrand, Durban, South Africa; 4 Department of Psychology, University of Miami, Miami, FL, United States; 5 Department of Psychological and Brain Sciences, Sexual, Reproductive, and Mental Health Disparities Program, Boston University, Boston, MA, United States

**Keywords:** South Africa, nurses, HIV, resiliency, intervention, randomized controlled trial, stress, burnout

## Abstract

**Background:**

South Africa has the largest HIV epidemic in the world; in KwaZulu-Natal Province, over 40.8% of adults aged 15 years and older are living with HIV. Despite this, South Africa is home to only 3% of the world’s health care workers. Nurses constitute the largest group of providers in South Africa and experience high levels of burnout, which can contribute to negative patient outcomes for people living with HIV, including reduced treatment adherence. Nurse-centered interventions that offset these effects are urgently needed.

**Objective:**

This study aims to test the feasibility and acceptability of an adapted resiliency intervention (Stress Management and Resiliency Training-Relaxation Response Resiliency Program) for professional nurses who provide care for people living with HIV in South Africa.

**Methods:**

In phase 1 (Human Research Ethics Committee [Medical] of the University of the Witwatersrand [220813, Johannesburg, South Africa] and the Massachusetts General Brigham Institutional Review Board [2022P002765, Boston, Massachusetts, United States]), we conducted 3 focus group discussions to solicit feedback on the lived experiences of stress, sources of stress, impact on job functioning, coping strategies, the proposed intervention, and recruitment strategies for nurses. These data informed adaptations to the intervention. In phase 2 (Human Research Ethics Committee [240106, Johannesburg, South Africa]; Massachusetts General Brigham Institutional Review Board [2024P001407, Boston, Massachusetts, United States]), we conducted a small proof-of-concept study (N=8) with preintervention and postintervention assessments, 6 intervention sessions with a nurse interventionist, and a qualitative exit interview. Following appropriate adaptations, we conducted a pilot randomized controlled trial (N=60) in which participants were randomized to the intervention or the control condition. The control condition received a one-time, 90-minute didactic stress management session. The intervention condition consisted of two 4-hour group skills-based sessions on the relaxation response, components of stress, recuperative sleep, mindful awareness, resilience, and social support. Sessions included practice-based exercises and videos to complement the intervention materials. Baseline, posttreatment (intervention only), and follow-up assessments, as well as qualitative exit interviews (n=15, intervention only), were conducted. Primary outcomes are feasibility (number screened, eligible, and enrolled; the number of treatment sessions and assessments completed in the intervention arm; assessment duration; and reasons for declining enrollment and prematurely leaving the trial) and acceptability (Client Satisfaction Questionnaire-8 and qualitative data).

**Results:**

The project is funded by the National Institute of Mental Health (R34MH126753; September 2022). As of October 2025, we have completed both the proof-of-concept study (n=8; February 2025) and the pilot randomized controlled trial (n=60; August 2025). Data analysis is in progress and is expected to be completed in August 2026.

**Conclusions:**

Structural changes are needed to ensure the well-being of health care providers; however, given that structural changes take time, money, and political capital to execute, we must develop interventions to support providers’ mental health while advocating for systematic change.

## Introduction

### Background

South Africa has the largest HIV epidemic in the world, with 7.9 million people infected and prevalence rates of 22.6% among females and 11.5% among males aged 15 to 49 years [1]. South Africa’s epidemic accounts for 19% of the global population of people living with HIV, 15% of new infections, and 11% of AIDS-related deaths worldwide [2]. KwaZulu-Natal Province is particularly affected; 40.8% of adults over age 15 years are living with HIV, and people living in this province have a 46% higher risk of infection than those outside the province [3]. South Africa has made considerable progress toward achieving the global 95-95-95 targets, but in 2023 only 83% of people living with HIV were on antiretroviral therapy (ART) and just 78% had suppressed HIV RNA [4]. Available interventions to support patient care engagement have not closed enough of the treatment gap, and innovative interventions to promote patient engagement are urgently needed, particularly those that support the needs of frontline providers, such as nurses.

Despite bearing nearly one-fifth of the global HIV burden, South Africa has only 3% of the world’s health care workers, a shortage caused by uneven geographical distribution of personnel, migration, and burnout [5,6]. Reported reasons for provider migration out of South Africa include dissatisfaction with pay, lack of advancement opportunities, and work-related stress [7-9]. Nurses constitute the largest group of health care providers in South Africa—81% of public sector health professionals in 2015 [10]. Yet, in 2019, there was an estimated shortage ranging from 26,000 to 62,000 nurses, and the shortage is expected to increase to 131,000 to 166,000 by 2030 [11]. Limited resources lead to overwhelming caseloads, resulting in burnout, low retention, chronic stress, hopelessness, and cynicism among nurses [9,12]. South African nurses are faced with chronic work overload and occupational stress [13-15], in part due to inadequate staffing, resources, and wages [9,14,16,17]. Since 2010, South Africa has increasingly adopted task-shifting as part of an effort to scale up its ART program; for nurses, this has meant an ever-greater task burden [18,19]. Household challenges can exacerbate work stressors, as the demands of nursing combined with traditional gender norms and family economic dependency contribute to chronic stress [20]. In a sample of South African hospital nurses, both personal and work-related stress were significantly associated with burnout [21]. Burnout—characterized by exhaustion, cynicism, and inefficiency—is a prolonged response to chronic emotional and interpersonal stressors [22] and is linked to job dissatisfaction and intention to leave [7,23].

Resource limitations and associated stress and burnout in nurses lead to negative patient outcomes that disrupt the HIV treatment cascade and compromise HIV outcomes. Patient engagement with HIV care (eg, attending appointments, adhering to ART, refilling prescriptions, managing side effects) is facilitated principally through nurse-patient relationships. Stress and burnout among nurses can negatively affect people living with HIV in a variety of ways, including (1) self-reported suboptimal care of and negative attitudes toward patients [24]; (2) decreased patient satisfaction [25,26]; (3) increased posthospitalization recovery time [26]; and (4) reduced ART adherence, HIV testing uptake, and prevention of mother-to-child transmission care due to experienced or anticipated negative provider interactions [27-29]. Patient perceptions of the patient-provider relationship are a core element of engagement and retention in HIV care [30,31]. Among first-time ART users, a strong patient-provider relationship has been found to be particularly important for maintaining adherence [32,33]; yet, patients have reported discrimination, stigmatization, and provider breaches of patient confidentiality at HIV clinics [34,35]. In general, patients prefer providers who have good communication skills and are empathetic, engaging, and validating [36]. These qualities build trust, which is positively associated with HIV care adherence [37]. When nurses who care for people living with HIV experience stress and burnout, patient-nurse relationships suffer and trust may be impacted; providers experiencing burnout report making treatment mistakes, yelling at patients, and rushing through diagnostic tests [38]. In South Africa, stress due to increased workload, staff shortages, and poor working conditions has been associated with nurse absenteeism [39], which further damages these relationships and cripples an underresourced health care system.

Even though HIV care nurses in South Africa experience particularly high levels of stress and burnout [9,13,14,16,40,41], there have been no systematic efforts to develop skills-based programs to help nurses manage and cope with the occupational-related and personal stressors that negatively affect patient outcomes. Stress reduction interventions have been used in a variety of medical settings across North America and have been found to decrease burnout and stress among nurses in oncology [42], pediatric intensive care units [43], and large health care organizations [44], as well as among nursing students [45]. In South Africa, an intervention that increases nurses’ resiliency may decrease stress, prevent burnout, and lead to improved patient engagement in HIV care and may be important to support nurse well-being. Resiliency is a “multidimensional construct that refers to the ability to maintain adaptation and effective functioning under significant adversity or challenging life conditions” [46], or stress. Skills that foster resilience include increasing social support, mastery (a sense of control over one’s life), optimism, acceptance, will to live, effective self-management, and generativity [47,48]. Resiliency interventions go beyond traditional stress management interventions by adding elements of positive psychology, which may teach individuals not only how to manage immediate stressors but also how to live purposefully, optimize health, and build “immunity” for better long-term stress management—key in this setting where widespread structural changes to decrease nurses’ stress are unlikely in the near future.

### Theoretical Framework

**Figure 1.**
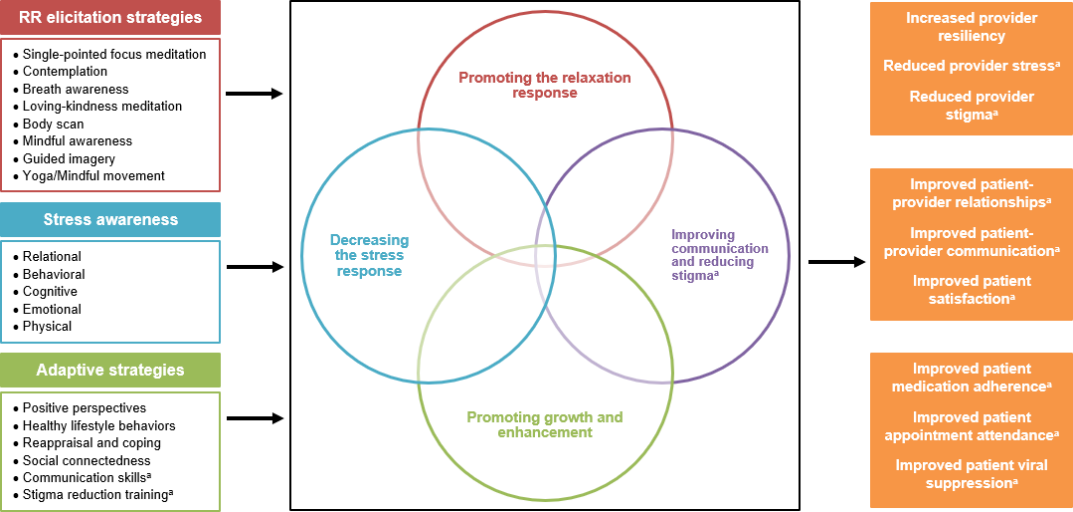
Overview of adapted Stress Management and Resiliency Training-Relaxation Response Resiliency Program model [46]. RR: relaxation response. ^a^Novel components added to the model.

The stress response, or the “fight-or-flight” response, is a set of physical changes in the body in response to demands ranging from major negative life events to small daily challenges. The goal of the Stress Management and Resiliency Training-Relaxation Response Resiliency Program (SMART-3RP) is to decrease the physiological, emotional, cognitive, and behavioral effects of the stress response by promoting the physiological, emotional, cognitive, and behavioral effects of the relaxation response (RR) [46]. To achieve these goals, the SMART-3RP focuses on three major areas: (1) eliciting the RR, (2) increasing stress awareness, and (3) promoting adaptive strategies. Eliciting the RR involves learning strategies to reduce muscle tension, breathing rate, heart rate, and blood pressure, with the aim of helping to regulate the cellular responses to stress. Decreasing stress reactivity involves increasing one’s awareness of being in the stress response (negative thoughts, emotions, physical reactions, and behaviors) and learning skills to change or alter these components (eg, cognitive restructuring). Adaptive strategies focus on learning and using different techniques to promote positive growth and self-efficacy in response to stress. Given high levels of community stigma toward people living with HIV [35] and patients’ desire for providers who have good communication skills and are empathetic, engaging, and validating [36], adaptations to the SMART-3RP were made by adding stigma reduction and communication skills as adaptive strategies. The intervention was further adapted based on phase 1 focus group discussion (FGD) feedback [49]. It is the learning and integration of these 3 core elements that enables individuals to adaptively cope with stressful situations and thus increase resiliency. The SMART-3RP model has been adapted and refined to reflect additional clinician-patient dynamics and interdependence (Figure 1; novel components added to the model are indicated with an asterisk).

### Study Aims

We aim to conduct a small proof-of-concept study (N=8) and a pilot randomized controlled trial (RCT; N=60) of the SMART-3RP, a skills-based intervention that has been tailored to enhance resiliency among professional nurses providing HIV care in the public sector in South Africa. We will test the feasibility and acceptability of all study procedures in preparation for a larger trial testing the efficacy and implementation strategies of the intervention. To assess feasibility, we tracked the number of participants screened, eligible, and enrolled; the number of treatment and assessment sessions completed; assessment duration; and reasons for declining enrollment and for prematurely leaving the trial. To assess acceptability, we used a measure of participant satisfaction and qualitative exit interview data. We hypothesize that the intervention and study procedures will be feasible and acceptable.

## Methods

### Adaptation of SMART-3RP Intervention

#### Focus Group Discussions

The first phase of this study collected formative, qualitative data via FGDs to inform the adaptation of the SMART-3RP for delivery to nurses providing HIV care in the public health sector in South Africa. The study was introduced to KwaZulu-Natal Provincial and eThekwini District health departments and to clinic managers and professional nurses from the selected clinics by study team members. Interested professional nurses were recruited from a public health care (PHC) facility in South Africa and were eligible if they (1) had provided HIV care in a public sector or partner organization health clinic at the level of professional nurse (ie, typically holding a Bachelor of Nursing degree and registration with the South African Nursing Council) for at least 12 months, (2) were able and willing to sign informed consent, and (3) were aged 18 years and older. A trained study team member without any supervisory power over the nurse interviewees facilitated 3 FGDs with a total of 14 nurse participants (one group of 4 and two groups of 5 participants each). FGDs lasted 50 to 90 minutes and were conducted in a private room.

A semistructured FGD guide (Multimedia Appendix 1) was developed by the study team based on a review of the existing literature and potential logistical issues that could impact intervention delivery. FGDs gathered information across three domains relevant to SMART-3RP intervention adaptation and implementation: (1) role of culture, perceptions, and experiences of stress; sources of stress (eg, occupational, trauma-related, structural); how stress impacts job functioning (specifically patient care); and current coping strategies; (2) recommendations about the intervention (eg, modules and content, number of sessions, session duration, program length, mode of delivery [virtual, in-person, hybrid approaches], and use of coaches); and (3) the most effective means to recruit professional nurses for a future clinical trial, including minimizing the possibility of coercion and ensuring nurses do not feel “blamed” for insufficient resilience. Most participants preferred an in-person, group format, believing this would enhance their interaction with one another as well as allow for open dialogue and deeper engagement with SMART-3RP material. Participants stressed the importance of (1) having an interventionist present to answer questions and facilitate engagement, (2) ensuring support from clinic management, which would communicate the value of the program to both staff and patients, and (3) normalizing discussions about stress. Participants were supportive of using texting platforms (eg, WhatsApp) to maintain connectedness with other participants and support ongoing skill use.

Changes to the content of the intervention were made based on the FGD results [49]. These included (1) integrating the normalization of stress and talking about stress, (2) discussing the importance of stigma awareness in patient care, (3) integrating an overview of practicing empathy and compassion in patient care while under high pressure, and (4) integrating examples with local imagery for relaxation exercises and adapting food and physical activity guidelines to match the guidelines for South Africa. Additionally, the intervention format was changed from 3 to 6, 1-hour each, in-person group sessions to accommodate the availability of staff to attend the sessions in the midst of a heavy workload and to avoid disrupting patient flow.

#### Proof-of-Concept Study

After conducting the FGDs and finalizing the study documents, we conducted a small proof-of-concept study (N=8) of the intervention, including baseline and posttreatment assessments, during which the adapted materials were simulated and tested. We conducted qualitative exit interviews (see Multimedia Appendix 2 for the interview guide) from 7 proof-of-concept study participants to explore their experiences in the study (eg, treatment content, length of sessions, scheduling/location of sessions, adherence to the skills covered in the sessions, and length of the overall intervention).

#### Refinement of the SMART-3RP Intervention

Additional changes were made to the intervention format and the protocol based on data from the proof-of-concept study and concurrent changes in health care delivery conditions in South Africa. Specifically, following the proof-of-concept study, South Africa experienced a large wave of layoffs among nurses providing care to people living with HIV due to reduced availability of funding that supports health care infrastructure in South Africa (eg, cuts to President’s Emergency Plan for AIDS Relief and US Agency for International Development); there was also concern that the grant would be terminated. To increase participant outreach and collect as much data as possible before a potential grant termination, the study team changed the format of the intervention to two 4-hour sessions and allowed for the participation of nurses who had recently been laid off but otherwise met the inclusion criteria.

### Pilot RCT

#### Characteristics of Study Population

We enrolled 60 professional nurses for the randomized pilot. Inclusion criteria were (1) having provided HIV care in a PHC facility (at the level of professional nurse) for at least 1 year; (2) being aged 18 years and older; and (3) being able and willing to sign informed consent. Exclusion criteria included participation in cognitive behavioral therapy and/or a mind-body intervention in the past year.

#### Recruitment and Enrollment

Participants were recruited from several PHC facilities (eg, primary health clinic, community health center, district hospital gateway clinic) and partner organizations that support the HIV program in the eThekwini District. By sampling from several PHC facilities in the eThekwini District of KwaZulu-Natal Province, we expected to recruit a representative sample of nurses providing HIV care in the primary health care sector. To identify eligible professional nurses, we consulted with KwaZulu-Natal Provincial Health Department managers, managers of partner organizations providing HIV care in eThekwini District, eThekwini District Health leadership, and PHC facility managers before the initiation of study procedures. Study participation was offered to all professional nurses meeting the inclusion criteria at each clinic until the target sample size was met.

#### Research Procedures

##### Informed Consent

A detailed informed consent form was reviewed with all participants by local study staff in a private setting. The consent form included an overview of all study procedures, information about potential risks and benefits of participation (including possible social harms associated with participation), and contact information of study team members.

##### Group Assignment

After completion of the small proof-of-concept study, the randomized pilot study (N=60 nurses) was conducted. Once participants were screened, they were asked to select their availability based on 4 possible dates, which were randomly assigned as intervention or control. The groups consisted of approximately 10 participants each.

##### Control Condition

The control condition received a 1-time, approximately 90-minute, interventionist-led didactic stress management session (with no interventionist follow-up). The session provided information on the 4-component model of stress and an “energy battery” exercise, which asked participants to reflect on stressors and things that offset stress (Textbox 1).

Textbox 1. **Stress management session content for control arm.**

- The science of mind-body medicine
- The stress response
- The flight-or-flight response
- Resiliency
- Cognitive behavioral therapy model of emotion
- The energy battery

##### Intervention Condition

The intervention condition consisted of two 4-hour interventionist-led group sessions. The sessions provided information on topics including the RR, the components of stress, recuperative sleep, mindful awareness, resilience, and social support (see Table 1). Sessions also included practice-based exercises (eg, breathing exercises and mindfulness exercises). Short videos were recorded and included in the sessions to complement the material on breathing, thought records (ie, a practical way to capture and examine thoughts), energy battery (ie, awareness of things that add to or deplete their personal battery), social support, mindfulness, and RR exercises. Participants were provided with a manual describing session content and audio recordings of RR exercises. Participants were encouraged to complete practice notes between and after sessions, which corresponded with intervention modules (eg, relaxation practice) to document their goals and progress.

**Table 1.** Adapted Stress Management and Resiliency Training-Relaxation Response Resiliency Program chapters and goals for the intervention arm.

Chapter title	Goals
Stress management and resiliency training	Introduce the SMART-3RP^a^ Introduce the Science of Mind-Body Medicine and its components: Stress Response, the Fight-or-Flight Response, and the RR^b^ Introduce Stress Management and Resiliency Training goals strategy Introduce RR-elicitation method: single pointed focus meditation, body and breath awareness, letter to yourself exercise Establish program and weekly goals
The RR	Introduce RR-elicitation method: autogenic training Introduce “MINIs”^c^ as a method to reduce tension and anxiety throughout the day Introduce the concept of appreciation Discuss recuperative sleep and sleep tips Introduce the Stress Warning Signals exercise
Stress awareness	Introduce mindfulness Introduce RR-elicitation method: mindful awareness MINI^c^ Learn the types of social support Identify what you need and what is feasible Discuss empathy and compassion
Mending mind and body	Introduce new RR-elicitation method: chair yoga Use strategies for applying mindful awareness in daily living Introduce the Pause, Breathe, Reflect, and Choose routine Reflecting on what’s important exercise
Creating adaptive perspective	Introduce new RR-elicitation method: loving kindness meditation Use cognitive reappraisal as a way to build adaptation Discuss negative automatic thoughts and thinking errors Learn about different coping styles: problem-solving and acceptance-based coping Explore the development of acceptance, an essential quality of acceptance-based coping
Promoting positivity	Introduce RR-elicitation method: idealized self Identify how positivity can increase resiliency in the long term Learn concepts and strategies for enhancing positivity Review SMART-3RP strategies learned Develop a plan for continuing to use program strategies Set goals for the future

a SMART-3RP: Stress Management and Resiliency Training-Relaxation Response Resiliency Program.

b RR: relaxation response.

c MINI: Mindfulness, Intention-setting, Nurture a hobby, Indulge in gratitude.

Participants also had the option of accessing a closed (eg, limited to group members from their cohort) WhatsApp group moderated by the interventionist. The interventionist initiated chats in between group sessions to provide support to participants in practicing stress management skills (eg, “What skills have you been able to practice?” and “Is there a skill that you need some support in using?”). In addition, the interventionist shared the recordings of the RR exercises and the short videos in the WhatsApp group on a weekly basis as a reminder to practice the RR exercises and maintain engagement with the participants.

All intervention sessions were delivered by a professional nurse who was trained on the intervention material and closely supervised by a clinical psychologist. Training sessions took place during the weeks leading up to and during the intervention delivery to review and practice the intervention content and to discuss strategies for delivering the intervention and facilitating group member comfort, participation, and cohesion. The interventionist offered 2 booster sessions, at approximately 1 month from the conclusion of session 2, to reinforce the intervention content and resiliency strategies. Participants were asked to sign a confidentiality statement prior to the inception of group sessions.

##### Fidelity

All sessions with the interventionist were audiotaped. Monitoring of the intervention will take into account both the interventionist’s adherence to the session material (eg, degree of coverage of session components) as well as competence [50] in delivering the material (eg, adherence to therapeutic skills, such as encouraging an environment of safety and encouraging discussion). Fidelity ratings will be completed using a checklist developed by the study team (see Multimedia Appendix 3). At least 10% of the sessions will be reviewed for fidelity by a study team member (LNT) with expertise in intervention strategies.

##### Retention

All study participants completed the Participant Locator Form, which collects their contact information, including social networks such as WhatsApp. Additionally, participants were asked to provide the contact information of 3 people to be contacted in the case that the research team failed to reach them. The research team tracked participants for follow-up visits and retention using phone calls and WhatsApp messages. The participants’ work schedules at the clinics were taken into consideration when scheduling phone calls and messages. Retention procedures were reviewed weekly by the Project Director and Principal Investigator.

##### Remuneration

Participants were reimbursed US $26 (R450) per study visit for their time, inconvenience, and transportation costs, as per South African Health Products Regulatory Authority guidelines.

##### Adverse Event Reporting

Adverse events are defined as harmful occurrences to participants, either study-related or non–study-related. Study staff were trained to make appropriate referrals for clinical care in consultation with the Principal Investigators. Adverse events meeting reporting criteria were reported as appropriate and were discussed during biweekly calls between the study Principal Investigator, recruiting site Principal Investigator, and Project Director.

##### Criteria for Discontinuation

Participants were informed that they could end study participation at any time if they desired and that withdrawal from the study would not impact their ongoing position at the health clinic facility.

### Data Collection

#### Assessment Instruments

Intervention participants were asked to complete 3 quantitative assessments (baseline assessment at enrollment [ie, prerandomization], a posttreatment assessment, and a final follow-up assessment at around the 3-month time point). Participants completed a qualitative exit interview (n=15) between the posttreatment and follow-up assessments. Control participants were asked to complete assessments at baseline and at approximately 3 months. Baseline assessments lasted approximately 45 minutes, and posttreatment and follow-up assessments each lasted approximately 35 to 40 minutes (see Table 2 for the list of instruments used at each assessment).

**Table 2.** Measures included at each major assessment.

Instruments	Baseline assessment	Posttreatment assessment	Follow-up assessment
Sociodemographics^a^	✓	—^b^	—
Resilience: Measure of Current Status (MOCS-A); Current Experiences Scale (CES) [51]	✓	✓	✓
Perceived stress: Perceived Stress Scale (PSS-10) [52,53]	✓	✓	✓
Burnout: Maslach Burnout Inventory Human Services Survey for Medical Personnel (MBI-HSS [MP]) [22]	✓	✓	✓
Absenteeism^a^	✓	✓	✓
Compassion fatigue: Professional Quality of Life Scale (ProQOL) Version 5 [54]	✓	✓	✓
Stigma: Measuring HIV Stigma and Discrimination Among Health Facility Staff: Standardized Brief Questionnaire [55]	✓	—	—
Anxiety: Generalized Anxiety Disorder (GAD-7) scale [56]	✓	✓	✓
Depression: Patient Health Questionnaire (PHQ-9) [57]	✓	✓	✓
Social support: Coping Orientation to Problems Experienced (COPE) Inventory [58]	✓	✓	✓
Optimism: Life Orientation Test – Revised (LOT-R) [59]	✓	✓	✓
Distress tolerance: Distress Tolerance Scale (DTS) [60]	✓	✓	✓
Acceptability: Client Satisfaction Questionnaire (CSQ-8) [61]^c^; Qualitative exit interviews^c^	—	✓	—
Adherence to Weekly Intervention Practice Exercises^a^^c^^d^	—	✓	✓

a Instrument developed by study team.

b Not available.

c Only for intervention arm.

d Will provide the research team with an understanding of whether intervention skills were implemented in nurse’s daily lives outside of the intervention setting and may be used to generate a rating of adherence to out-of-session practice guidelines during study analyses.

#### Qualitative Interview

Qualitative exit interviews were conducted with 15 participants in the intervention arm between the posttreatment and follow-up assessments to explore acceptability. Interviews covered overall experiences with the SMART-3RP intervention, as well as feedback regarding the content and format of each individual intervention session (see Multimedia Appendix 2 for the interview guide).

### Data Analysis

The sample size (N=60) was chosen based on studies of similar scope (eg, other pilot behavioral trials funded via the same mechanism) and established guidelines for pilot behavioral studies to establish feasibility [62-64].

To evaluate feasibility as a coprimary outcome, we will assess the number of nurse participants screened, eligible, and enrolled (ie, whether >70% of those screened enroll/agree to participate); the number of treatment sessions (ie, whether >70% of participants randomized to the intervention complete at least 2 out of 3 sessions) and assessment visits completed (ie, whether >70% of all participants complete at least 2 out of 3 assessments) in the intervention arm; assessment duration; and reasons for declining enrollment and prematurely leaving the trial. Acceptability (second primary outcome) will be evaluated using the Client Satisfaction Questionnaire (CSQ-8) [61] and qualitative exit interview data among intervention arm participants. Scores on the CSQ-8 will be categorized into 4 levels of satisfaction: ‘‘poor’’ (score 8‐13), ‘‘fair’’ (score 14‐19), ‘‘good’’ (score 20‐25), and ‘‘excellent’’ (score 26‐32). All qualitative exit interview data will be explored for categories and themes around intervention acceptability. Qualitative data will be analyzed using content analysis, facilitated by the Dedoose software [65], to organize data and facilitate analyses. Two study team members will independently review the transcripts to generate an overarching thematic framework for data interpretation, in which major and minor themes are identified. Thematic frameworks will then be compared for consistency, and discrepancies will be discussed until agreement is reached. Data will be reexamined, messages will be extracted and highlighted, and ongoing discussion between coders will allow for further theorizing and making interconnections between research questions, coding categories, and raw data [66].

With respect to exploratory evaluations of quantitative data and secondary outcomes, we expect to compare each outcome between groups using mixed model regression analyses, with participant ID as a random effect. Fixed covariates will include assessment time point (baseline, posttreatment assessment, and 3-month follow-up) and years working in HIV care. Other covariates, such as education, age, and baseline levels of stress, anxiety, and depressive symptoms, and clinic characteristics (eg, clinic size, availability of equipment, and urban vs peri-urban) will also be considered. We will consider exploratory mediational and moderational analyses, based on procedures outlined by Hayes [67]. We will examine patterns in missing data to see if missingness is related to measured participant characteristics. We will adjust for missing data using multiple imputation, as appropriate. We will also adjust for multiple comparisons using the method of Hothorn et al [68].

### Data Management

Quantitative data were collected by trained study staff and entered into REDCap (Research Electronic Data Capture) data management system using encrypted tablets and computers. All tablets were encrypted to the highest standard. Qualitative data were collected by trained study staff, and deidentified transcripts will be coded using Dedoose software [65] to facilitate analyses. Data files were sent to the investigators from the site via secure file transfer.

### Ethical Considerations

All research was performed in accordance with the Declaration of Helsinki and approved by local and international ethics committees. Ethics approval for the study protocol was obtained from the Human Research Ethics Committee (Medical) of the University of the Witwatersrand (240106, Johannesburg, South Africa Provincial Department of Health, the Massachusetts General Brigham Institutional Review Board (2024P001407, Boston, Massachusetts, United States), and letters of support obtained from the District and supporting organizations. Study updates and final findings will be communicated to study participants. All data shared between study sites are fully deidentified. Consent procedures complied with Institutional Review Board requirements by ensuring that participants had the option to opt out without adverse consequences (ie, affecting their employment). Study updates and deidentified findings will also be shared at Data and Safety Monitoring Board (DSMB) meetings organized by the Principal Investigator and the Community Advisory Board meetings convened quarterly at Wits Match Research Unit. Lastly, data will also be presented at national and international scientific meetings and conferences and published in peer-reviewed journals.

We have convened a DSMB comprised of individuals from South Africa with relevant expertise to this study; meetings are conducted in accordance with a DSMB manual outlining the responsibilities of the DSMB, DSMB meeting procedures, and templates for DSMB reports (developed in collaboration with the DSMB members). The investigators are responsible for compiling and submitting annual reports. The DSMB members have reported no conflicts of interest, and their membership was approved by the funder. The DSMB has met twice to allow for additional, external review of study procedures and participant safety. No significant safety concerns were identified. The charter for this study can be requested from the corresponding author.

## Results

Eight participants were recruited for the proof-of-concept trial, of whom 100% were female, 75% (n=6) identified as Black, and 75% (n=6) had at least 6 years of experience providing HIV care. All screened participants enrolled, and 100% (N=8) of participants completed at least half of the intervention sessions and at least 2 out of 3 assessments. With respect to acceptability, satisfaction with the intervention was rated as “excellent” on the CSQ-8 (mean 29.25, SD 1.98). Exit interview data highlighted the need for flexibility in intervention delivery (eg, offering multiple opportunities to attend sessions and online content) to support feasibility. Exit interview data also indicated that group sessions were the only space dedicated to the nurses’ well-being, that awareness of the stress experiences of their colleagues led to increased compassion for colleagues, and that relaxation strategies helped support positive engagement with patients during challenging clinical encounters.

Sixty participants have been enrolled in the RCT. As described above, due to concerns around funding and possible study termination, all participants have completed intervention delivery and follow-up assessments. We are currently in the process of data analysis and offering control participants the intervention content. We expect data analysis to be completed in approximately August 2026, at which time we expect to present data consistent with the analyses presented in the *Data Analysis* section.

## Discussion

### Expected Outcomes and Future Directions

Nurses providing HIV care in South Africa manage substantial amounts of stress arising from multiple sources, including the aftereffects of the COVID-19 pandemic and structural challenges associated with the nursing profession. High levels of stress can negatively impact the health of nurses themselves and may lead to negative patient outcomes via reduced patient satisfaction, reduced treatment adherence, and reduced testing uptake. There is a dearth of evidence as to how best to support the mental health of nurses, including the feasibility and acceptability of asking overburdened health care providers to take part in multisession behavioral interventions. We hypothesize that we have developed a feasible and acceptable intervention that has the potential to demonstrate meaningful changes for managing stress among nurses in a resource-limited setting. Next steps will include the RCT data analyses and reporting of findings and may include a larger trial to examine effectiveness and implementation outcomes.

### Conclusion

Structural changes are needed to ensure the well-being of health care providers across all contexts. However, given that structural changes take time, money, and political capital to execute, we must develop interventions to support mental health while advocating for systematic change.

## Supplementary material

10.2196/79777 Multimedia Appendix 1 Semistructured focus group discussion guide.

10.2196/79777 Multimedia Appendix 2 Qualitative semistructured exit interview guide.

10.2196/79777 Multimedia Appendix 3 Fidelity rating checklist for Stress Management and Resiliency Training-Relaxation Response Resiliency Program.

10.2196/79777 Multimedia Appendix 4 National Institute of Mental Health review summary statement.

10.2196/79777 Checklist 1 SPIRIT checklist.

## Abbreviations

ART: antiretroviral therapy
CSQ-8: Client Satisfaction Questionnaire
DSMB: Data and Safety Monitoring Board
FGD: focus group discussion
PHC: public health care
RCT: randomized controlled trial
REDCap: Research Electronic Data Capture
RR: relaxation response
SMART-3RP: Stress Management and Resiliency Training-Relaxation Response Resiliency Program
SPIRIT: Standard Protocol Items: Recommendations for Interventional Trials
