# Supplementary material for: A Resiliency Intervention to Support Nurses Engaged in the Provision of HIV Care in KwaZulu-Natal, South Africa: Protocol for a Pilot Randomized Controlled Trial
Source: JMIR Res Protoc. 2026 Jun 25;15:e79777. doi: 10.2196/79777 (PMC13304968; doi:10.2196/79777)
Supplement: Multimedia Appendix 1 [file resprot-v15-e79777-s001.pdf]

| FOCUS GROUP NUMBER: | DD/MMM/YYYY): | START TIME: | END TIME: | INTERVIEWER INITIALS: |
|---|---|---|---|---|
| | | | | |

# Developing a resiliency intervention to support nurses engaged in the provision of HIV care (PHASE 1)

## Reminder to interviewers:

Questions in the left-hand column will be asked of all participants, while questions in the right-hand column are probes, and are to be asked only to help the participant describe their experiences **or** if the participants do not bring up these topics spontaneously.

## Interview script:

Welcome everyone and thank you for coming and agreeing to participate. Before we begin, I would like to explain a few important issues that we all have to follow.

- First everyone has agreed to the informed consent, yes?
- Second, I would like to remind you that this discussion will be audio recorded. Is everyone okay with this?
- I also want to remind you to maintain the confidentiality of the group. Please do not share things we discuss today outside of the group. We will use numbers to identify each other during the group discussion today. As a reminder, I will use a digital recorder to record our conversation.
- In addition, please remember to take turns when you are speaking so that everyone has an opportunity to participate in the discussion.

In today's discussion we are going to talk about levels of stress experienced by nurses who are providing HIV care. We are hoping to understand the experience of stress among nurses who provide HIV care in South Africa more fully so that we may develop an intervention that supports the emotional well-being of these nurses.

As a reminder, your participation is strictly voluntary and not linked to your employment in any way. Your responses will not be shared with your clinic supervisors. We will not identify you or your responses individually. You are not required to answer my questions, and you may decline to answer any questions that make you uncomfortable. As a reminder, we ask that you keep the content of our interview confidential, meaning, we ask that you not share what was discussed with people who are not part of this group.

Do you have any questions before we begin the interview? Now let us start our discussion.

**(TURN ON DIGITAL RECORDER)**

I am [INTERVIEWER NAME] interviewing participant [FOCUS GROUP #] on [DATE] [START TIME].

| | **Topics and Main Questions** | **Probes** |
|---|---|---|
| | **(1) General perceptions of stress** | |
| 1.1 | I am going to begin by asking you some general questions about stress.<br>What does stress mean to you?<br>What does resilience mean to you? (IF NEEDED: By resilience, I mean an individual's ability to manage chronic stress in positive ways.) | |
| 1.2 | How does your culture think about stress? By culture, I mean the values and beliefs of your social group(s), including your community, religious, and other social groups with whom you identify.<br><br>How do people within your cultural group(s) manage stress? | |

| | | |
|---|---|---|
| 1.3 | How do you typically manage stress? | How well do these strategies work?<br>When do these strategies work best?<br>When are these strategies least likely to work? |
| 1.4 | What role does mental health counseling play in helping nurses manage their stress? | |
| 1.5 | What are some sources of stress? | What things cause you to experience stress?<br>How does stress affect you? |
| | **<u>(2) Occupational stress</u>** | |
| 2.1 | Please tell me about how some of the structural issues nurses face impact your stress. By structural issues, I mean issues related to staffing, compensation, and other similar issues. | What support is available to help you cope with these issues?<br><br>What is needed to help you cope with these issues? |
| 2.2 | How does stress impact you at work? | How does stress impact the way you relate to your colleagues?<br><br>How does stress influence how you feel about your job? |
| 2.3 | How does stress impact your ability to care for your patients, or clients? | Tell me what would make you better able to care for your patients or clients.<br>What support do nurses need in order to care for patients or clients? |
| 2.4 | Tell me how COVID-19 has impacted your level of stress. | How has COVID-19 impacted your ability to care for your patients or clients as you would like? |

| | (3) Feedback on proposed intervention | |
|---|---|---|
| 3.1 | We have developed a programme to help people better manage stress and cope with challenging circumstances, and I would like to understand how this programme could work for you. Some parts of the programme would involve learning skills from someone trained in the programme.<br><br>This could be done in person or by using a computer or a phone. Please tell me how you would like to learn the skills. | Why? |
| 3.2 | How much time in a given week might you have to learn stress management skills? | What would help you practice these skills? |
| 3.3 | Our programme currently has eight weeks of 90-minute sessions of teaching and skill practice.<br><br>How do you feel about this? | How would this need to change to make it work for you?<br><br>Tell me how you feel about the number of weeks (eight).<br><br>Tell me how you feel about 90-minute teaching and practice sessions. |
| 3.4 | How would you feel about learning the skills via a video that you watch when you have time? | |
| 3.5 | How would you feel about coaching sessions with the person who teaches the skills to ask questions about the skills and to help you practice them? | |

| | | |
|---|---|---|
| 3.6 | We would like to teach a group of nurses the skills in a group. Please tell me how you feel about this. | How would you like to connect with other nurses learning these skills?<br><br>Why? |
| 3.7 | How would you feel about using social media, such as WhatsApp, to connect with other nurses about managing stress? | What are the advantages of this strategy?<br><br>What are the disadvantages? |
| 3.8 | How might we introduce this programme to nurses? | What would make it easier to participate? |
| 3.9 | What do we need to consider so nurses do not feel blamed for challenges managing stress? | |
| 3.10 | We plan to recruit from various clinics for our programme. How can we recruit in a way that does not create pressure for nurses to participate? | |
| | **(4) Conclusion** | |
| 4.1 | Thank you all for sharing your ideas and opinions with me. What have I not asked about that may be important to consider as we work to design a programme to help nurses manage their stress?<br><br>**END time for the discussion** | |

Thank you very much for participating today. We have learned a lot from everyone. We appreciate your openness and we will maintain confidentiality of this discussion as discussed in the consent. Thank you again.
