# Supplementary material for: A Resiliency Intervention to Support Nurses Engaged in the Provision of HIV Care in KwaZulu-Natal, South Africa: Protocol for a Pilot Randomized Controlled Trial
Source: JMIR Res Protoc. 2026 Jun 25;15:e79777. doi: 10.2196/79777 (PMC13304968; doi:10.2196/79777)
Supplement: Multimedia Appendix 2 [file resprot-v15-e79777-s002.pdf]

Qinisa Study – Phase 2
Qualitative Exit Interview

**Date (DD/MM/YYYY)**	*__ __ / __ __ / __ __ __ __*
**Time Start**	*__ __ : __ __ AM / PM*
**Time End**	*__ __ : __ __ AM / PM*
**Interviewer Initials**	

Reminder to interviewers:

Questions in the left-hand column will be asked of all participants, while questions in the right-hand columns are probes, and are to be asked only to help the participant describe her experience **or** if the participant does not bring up these topics spontaneously.

Exit interview script:

Hello, my name is [INTERVIEWER NAME]. I am from MatCH Research Unit (MRU). We are conducting interviews with nurses who participated in our stress management and resiliency-enhancing programme to better understand how they felt about it. We will use your feedback to make improvements to the programme for the future. No programme is perfect; please do not worry about hurting our feelings. If you found that something was not to your liking, please let us know so we can make the programme better for nurses who care for persons living with HIV in the public sector in South Africa.

As a reminder, you are not required to answer my questions, and you may skip any questions that make you uncomfortable. If you decide that you no longer want to participate in this interview, you can still get medical care at your clinic. As a reminder, I will use a digital recorder to record our conversation.

Do you have any questions before we begin the interview?

(TURN ON DIGITAL RECORDER)

I am [INTERVIEWER NAME] interviewing participant [PARTICIPANT #] on [DATE] at [START TIME].

Topics and Main Questions	Probes
**(1) Overall experience**	
I am going to begin by asking you about your experience in the study. How would you describe your overall experience attending the sessions?	What worked well for you?<br><br>What did you find most difficult?<br><br>Tell me how you felt about the <u>length</u> of the sessions, meaning how long each session lasted.<br><br>Tell me how you felt about the <u>number</u> of sessions, meaning how many times we met.<br><br>Would you be interested in booster sessions in the future? Why or why not? [Interviewer to define "booster session" for participant.]
What expectations did you have about the group sessions?<br><br>What could we/you have done differently to make your experience in the study better?	How did your expectations compare to your actual experience?<br><br>What, if anything, did you get from attending the sessions that you did <u>not</u> expect to get?<br><br>What was going on in your life that made it hard for you to participate (if applicable)?

Please describe your experience practicing the skills learned in sessions (e.g., relaxation exercises).<br><br>Would you recommend this study to other nurses?	How frequently did you meet your goals for practicing the skills you learned in the group?<br><br>What was going on in your life that made it hard for you to practice (if applicable)? What helped you practice?<br><br>Why or why not?
How did the programme and programme content affect your ability to manage stress at work?	
What did you think of the person running your group?	What personal characteristics are important to you in a group leader? Why?
**(2) Relaxation response and sleep**	
Thank you sharing your overall experiences with me. I would now like to hear your opinion on the skills presented in sessions. You may recall that we reviewed a number of topics, including the relaxation response (RR). We also practiced various exercises to elicit the RR (e.g., breathing exercises).<br><br>We also discussed how getting quality sleep can help to promote the relaxation response.	Did you find this topic helpful? Why/why not?<br><br>How did learning the information/skills change your behavior, if at all? How do you use the skills?<br><br>Please provide any suggestions for improving session content (e.g., change of format). How could this topic be improved?<br><br>Did you find this topic helpful? Why/why not?

	How did learning the information/skills change your behavior, if at all? How do you use the skills?<br><br>Please provide any suggestions for improving session content (e.g., change of format). How could this topic be improved?
**(3) Stress and positive perspective**	
We also discussed the components of stress, which include emotional, behavioral, physical, and cognitive aspects.	Did you find this topic helpful? Why/why not?<br><br>How did learning the information/skills change your behavior, if at all? How do you use the skills?<br><br>Please provide any suggestions for improving session content (e.g., change of format). How could this topic be improved?
**(4) Mindful awareness**	
We also talked about the concept of mindful awareness (or observing your thoughts/emotions in the moment).	Did you find this topic helpful? Why/why not?<br><br>How did learning the information/skills change your behavior, if at all? How do you use the skills?<br><br>Please provide any suggestions for improving session content (e.g., change of format). How could the topic be improved?

**(5) Resilience**	
As you know, the content of this programme was meant to help participants increase their resilience (or the ability to adapt and respond to stress in healthy ways) as nurses providing HIV care.<br><br>Tell me about any changes in your level of resilience or how well you can manage stress during the study. In what way(s), if at all, did it change?	Tell me how you feel about maintaining resilience in the future?<br><br>What do you believe will be most important in maintaining resilience? Which topic(s) / skill(s) from the programme will help you the most?
**(6) Social support**	
We also discussed the different types of social support that you might give and receive – including emotional, informational, tangible, self-esteem, and belonging support. We also talked about identifying and feeling comfortable asking for specific social support needs.	Did you find this topic helpful? Why/why not?<br><br>How did learning the information/skills change your behavior, if at all? How do you use the skills?<br><br>Please provide any suggestions for improving session content (e.g., change of format). How could the topic be improved?
**(7) Conclusion**	
What else you would like to share with me about your experiences with the programme?	What do you think was missing from the programme?
I want to thank you for your time today and for participating in our study. Your answers to our questions help us understand how to improve our programme for other nurses providing HIV care.	
