# Supplementary material for: A Resiliency Intervention to Support Nurses Engaged in the Provision of HIV Care in KwaZulu-Natal, South Africa: Protocol for a Pilot Randomized Controlled Trial
Source: JMIR Res Protoc. 2026 Jun 25;15:e79777. doi: 10.2196/79777 (PMC13304968; doi:10.2196/79777)
Supplement: Multimedia Appendix 3 [file resprot-v15-e79777-s003.pdf]

The Qinisa Study Phase 2

Fidelity / Competency Rating Form (Intervention)

Chapter 1: Stress Management and Resiliency Training

Study Site:
Date of Rating:
Name of Rater:

Participant Group #:
Date of Session:
Name of Group Leader (Interventionist):

Group Leader Fidelity (Chapter Components)	Poor / no coverage	Fair coverage	Good coverage	Very Good coverage	Excellent coverage
1. Resiliency and the Relaxation Response	0	1	2	3	4
2. Setting SMART Goals	0	1	2	3	4
3. Tips for Developing a Consistent Practice	0	1	2	3	4
4. The Stress Response	0	1	2	3	4
5. The Fight-or-Flight Response	0	1	2	3	4
6. The RR	0	1	2	3	4
7. Building Resiliency	0	1	2	3	4
8. Learning How to Breathe Again	0	1	2	3	4
9. RR Practice: Simple Breath Awareness	0	1	2	3	4
10. The Energy Battery	0	1	2	3	4
11. Exercise: Your Energy Battery	0	1	2	3	4
12. Exercise: Body Awareness	0	1	2	3	4
13. Exercise: Single-Pointed Focus Meditation	0	1	2	3	4
14. Exercise: Letter to Yourself	0	1	2	3	4
15. Guided Meditations and Apps	0	1	2	3	4

Total Fidelity Score: _____ / 60

Notes:

The Qinisa Study Phase 2
Fidelity / Competency Rating Form (Intervention)
Chapter 2: The Relaxation Response

Study Site: | Participant Group #:
Date of Rating: | Date of Session:
Name of Rater: | Name of Group Leader (Interventionist):

	Group Leader Fidelity (Chapter Components)	Poor / no coverage	Fair coverage	Good coverage	Very Good coverage	Excellent coverage
1.	Tips for Developing a Consistent RR Practice	0	1	2	3	4
2.	RR Practice: Autogenic Training	0	1	2	3	4
3.	Appreciations	0	1	2	3	4
4.	Recuperative Sleep	0	1	2	3	4
5.	Sleep Tips	0	1	2	3	4
6.	The MINI	0	1	2	3	4
7.	RR Practice: The MINI	0	1	2	3	4
8.	Additional Minis	0	1	2	3	4
9.	Exercise: Stress Warning Signals	0	1	2	3	4

Total Fidelity Score: _____ / 36

Notes:

The Qinisa Study Phase 2
Fidelity / Competency Rating Form (Intervention)
Chapter 3: Stress Awareness

Study Site:
Date of Rating:
Name of Rater:

Participant Group #:
Date of Session:
Name of Group Leader (Interventionist):

	Group Leader Fidelity (Chapter Components)	Poor / no coverage	Fair coverage	Good coverage	Very Good coverage	Excellent coverage
1.	Appreciations	0	1	2	3	4
2.	Mindful Awareness	0	1	2	3	4
3.	RR Practice: Mindful Awareness MINI	0	1	2	3	4
4.	Social Support Domains	0	1	2	3	4
5.	Exercise: The Social Support Diagram	0	1	2	3	4
6.	Exercise: Identifying What You Need and What is Feasible	0	1	2	3	4
7.	Healing States of Mind: Empathy and Compassion	0	1	2	3	4
8.	Exercise: Empathy/Relating to Others	0	1	2	3	4
9.	Empathy and Compassion in Client Care When Under Stress	0	1	2	3	4

Total Fidelity Score: _____ / 36

Notes:

The Qinisa Study Phase 2
Fidelity / Competency Rating Form (Intervention)
Chapter 4: Mending Mind and Body

Study Site:
Date of Rating:
Name of Rater:

Participant Group #:
Date of Session:
Name of Group Leader (Interventionist):

	Group Leader Fidelity (Chapter Components)	Poor / no coverage	Fair coverage	Good coverage	Very Good coverage	Excellent coverage
1.	Appreciation	0	1	2	3	4
2.	Awareness of Movement	0	1	2	3	4
3.	RR Practice: Yoga	0	1	2	3	4
4.	Bringing it All Together: Pause, Breathe, Reflect, Choose	0	1	2	3	4
5.	Exercise: Reflecting on What’s Important	0	1	2	3	4

Total Fidelity Score: _____ / 20

Notes:

The Qinisa Study Phase 2
Fidelity / Competency Rating Form (Intervention)
Chapter 5: Creating an Adaptive Perspective

Study Site:
Date of Rating:
Name of Rater:

Participant Group #:
Date of Session:
Name of Group Leader (Interventionist):

	Group Leader Fidelity (Chapter Components)	Poor / no coverage	Fair coverage	Good coverage	Very Good coverage	Excellent coverage
1.	Appreciation	0	1	2	3	4
2.	RR Practice: Loving Kindness Meditation	0	1	2	3	4
3.	Negative Automatic Thoughts	0	1	2	3	4
4.	Thinking Errors	0	1	2	3	4
5.	Beliefs Give Way to Emotions	0	1	2	3	4
6.	Exercise: Coping Log, Part 1	0	1	2	3	4
7.	Coping Strategy: Creating Adaptive Perspectives	0	1	2	3	4
8.	Coping Strategy: Challenging Unhelpful Thoughts	0	1	2	3	4
9.	Exercise: Coping Log, Part 2	0	1	2	3	4
10.	Coping Strategy: Problem-Solving vs. Acceptance	0	1	2	3	4
11.	Determining the Appropriate Coping Strategy	0	1	2	3	4
12.	Exercise: Problem-Solving	0	1	2	3	4

Total Fidelity Score: _____ / 48

Notes:

The Qinisa Study Phase 2
Fidelity / Competency Rating Form (Intervention)
Chapter 6: Promoting Positivity

Study Site: Participant Group #:
Date of Rating: Date of Session:
Name of Rater: Name of Group Leader (Interventionist):

	Group Leader Fidelity (Chapter Components)	Poor / no coverage	Fair coverage	Good coverage	Very Good coverage	Excellent coverage
1.	Appreciation	0	1	2	3	4
2.	RR Practice: Idealized Self	0	1	2	3	4
3.	Exercise: The Energy Battery, Take 2	0	1	2	3	4
4.	Exercise: From Pessimism to Optimism	0	1	2	3	4
5.	Summary of Strategies	0	1	2	3	4
6.	Tips for Staying Resilient	0	1	2	3	4

Total Fidelity Score: _____ / 24

Notes:

The Qinisa Study Phase 2
Fidelity / Competency Rating Form (Intervention)
Session 1: Competency Rating Form

Study Site:
Date of Rating:
Name of Rater:

Participant Group #:
Date of Session:
Name of Group Leader (Interventionist):

Group Leader Competence		Not at all	Somewhat	Good	Very Good	Excellent
1.	Established / maintained a safe and welcoming environment	0	1	2	3	4
2.	Modeled good listening skills and encouraged participant discussion	0	1	2	3	4
3.	Addressed emotions and patient concerns in a timely fashion	0	1	2	3	4
4.	Assertively guided group members through the curriculum	0	1	2	3	4

Total Competence Score: _____ / 16

Notes:

The Qinisa Study Phase 2
Fidelity / Competency Rating Form (Intervention)
Session 2: Competency Rating Form

Study Site:
Date of Rating:
Name of Rater:

Participant Group #:
Date of Session:
Name of Group Leader (Interventionist):

Group Leader Competence		Not at all	Somewhat	Good	Very Good	Excellent
1.	Established / maintained a safe and welcoming environment	0	1	2	3	4
2.	Modeled good listening skills and encouraged participant discussion	0	1	2	3	4
3.	Addressed emotions and patient concerns in a timely fashion	0	1	2	3	4
4.	Assertively guided group members through the curriculum	0	1	2	3	4

Total Competence Score: _____ / 16

Notes:
