# Supplementary material for: A Resiliency Intervention to Support Nurses Engaged in the Provision of HIV Care in KwaZulu-Natal, South Africa: Protocol for a Pilot Randomized Controlled Trial
Source: JMIR Res Protoc. 2026 Jun 25;15:e79777. doi: 10.2196/79777 (PMC13304968; doi:10.2196/79777)
Supplement: Multimedia Appendix 4 [file resprot-v15-e79777-s004.pdf]

SUMMARY STATEMENT
(Privileged Communication)

PROGRAM CONTACT:
Christopher Gordon
240-627-3867
cgordon1@mail.nih.gov

Release Date: 08/03/2021
Revised Date:

Application Number: 1 R34 MH126753-01A1
Formerly: 1R34MH126753-01

Principal Investigator

PSAROS, CHRISTINA

Applicant Organization: MASSACHUSETTS GENERAL HOSPITAL

Review Group: HIBI
HIV/AIDS Intra- and Inter-personal Determinants and Behavioral Interventions Study Section
AIDS - EXP. REV.

Meeting Date: 07/12/2021
Council: OCT 2021
Requested Start: 09/01/2021
RFA/PA: PA20-141
PCC: 9A-ASGC

Project Title: Developing a resiliency intervention to support healthcare workers engaged in the provision of HIV care
SRG Action: Impact Score:34 Percentile:18 +
Next Steps: Visit https://grants.nih.gov/grants/next_steps.htm
Human Subjects: 30-Human subjects involved - Certified, no SRG concerns
Animal Subjects: 10-No live vertebrate animals involved for competing appl.
Gender: 1A-Both genders, scientifically acceptable
Minority: 5A-Only foreign subjects, scientifically acceptable
Age: 3A-No children included, scientifically acceptable

Project Year	Direct Costs Requested	Estimated Total Cost
1	125,000	170,339
2	200,000	272,542
3	125,000	170,339
TOTAL	450,000	613,219

ADMINISTRATIVE BUDGET NOTE: The budget shown is the requested budget and has not been adjusted to reflect any recommendations made by reviewers. If an award is planned, the costs will be calculated by Institute grants management staff based on the recommendations outlined below in the COMMITTEE BUDGET RECOMMENDATIONS section.

1R34MH126753-01A1 Psaros, Christina

RESUME AND SUMMARY OF DISCUSSION: This application proposes to adapt the Relaxation Response Resiliency Program (3RP), a proven intervention that seeks to elicit the relaxation response, increase stress awareness and promote adaptive strategies to reduce physical responses to stress (e.g., heart rate, blood pressure), for use with nurses providing care to persons living with HIV in South Africa through information gained from several qualitative focus group discussions with nurses. Then the team will conduct a pilot RCT of the adapted intervention with 60 nurses in South Africa to test the feasibility and acceptability of study procedures. Both patient- and clinic-level data will also be gathered to prepare for a R01 to test the efficacy of the intervention. Nurse stress and burnout can lead to negative outcomes for persons living with HIV, and this effort to reduce stress in HIV care nurses in South Africa, a setting with very high HIV prevalence, has the potential to have a substantial public health impact. The study team is very strong and they have been very responsive to the concerns that were raised in the prior review of this application. The sample size was increased, team members were added, and references to effect sizes were removed. Some members were concerned that proposed intervention would have little sustained impact since it only addresses individual factors (HIV care delivered by nurses) and does not address any of the structural factors that increase the nurses' stress. It was also noted that other members of the staff in the clinics could be experiencing stress and it was not clear why the intervention was limited to the nurses. There were other minor concerns about the proposed survey of the patients. Despite these concerns, most members of the committee focused on the lack of interventions for health care providers in South Africa and, therefore, the committee's overall enthusiasm for this much improved resubmission remained high.

DESCRIPTION (provided by applicant): South Africa (SA) has the largest HIV epidemic in the world, with 7.2 million people infected and a prevalence of 18.8% among adults ages 15-49. Nurses constitute the largest group of providers in SA's healthcare system and are frontline treaters in HIV care. They experience particularly high levels of burnout resulting from resource limitations, chronic work overload, and occupational stress; inadequate staffing, resources, and wages; increased task-shifting work to nurses; interpersonal conflict; and HIV stigma, including stigma by association. These stressors are worsened in the context of the COVID-19 pandemic. Provider stress and burnout leads to negative patient outcomes for persons living with HIV (PLWH), including: (1) self-reported suboptimal care and attitudes toward patients; (2) decreased patient satisfaction; (3) increased post- hospitalization recovery time; and (4) reduced adherence to highly active antiretroviral therapy (HAART) and HIV testing uptake as a result of experienced or anticipated negative patient-provider interactions. Patient- provider relationships are a core element of engagement and retention in HIV care; patients prefer providers who have good communication skills, and are empathetic, engaging, and validating, behaviors that are difficult to emulate in the context of high stress and burnout. Interventions that foster resilience can minimize the negative impact of work-related stress; however, despite decades of research into individual, patient-level factors impacting HIV care engagement, no known interventions have systematically attempted to support the wellbeing of nurses in SA by providing them with skills to more effectively manage and cope with stressors that negatively and directly impact patient outcomes. The objective of this application is to adapt the Relaxation Response Resiliency Program (3RP) to the needs of nurses providing HIV care in the public sector in SA to build resilience and reduce stress, and ultimately improve patient-related outcomes. We propose the following specific aims as part of this formative feasibility and acceptability study: (1) to explore the ways in which the 3RP requires adaptation to meet the needs of nurses providing HIV care in the public sector in SA, based on data collected from qualitative focus group discussions, and to adapt the intervention accordingly, and (2) after a small proof-of-concept study, to conduct a randomized pilot of nurses providing HIV care in the public sector in SA to test the feasibility and acceptability of all study procedures. The proposed work will contribute to our understanding of how to offset high stress and burnout among HIV care nurses in

SA's public sector, a setting with the world's most significant HIV burden, where nurses are the frontline treaters. These data will inform a larger, multi-site, randomized efficacy trial looking at the impact of the intervention on the wellbeing of nurses, as well as on patient outcomes (e.g., successful engagement in HIV care, patient-nurse trust, satisfaction with care, and viral suppression) in public health sector clinics nationwide. Ultimately, we hope to develop an intervention with applicability to other providers in other resource-limited settings.

PUBLIC HEALTH RELEVANCE: Despite many years of research that have focused almost exclusively on patient-level barriers to care (despite the importance of positive patient-provider relationships in HIV care engagement), there are still significant gaps in the number of persons living with HIV (PLWH) who are fully engaged in care, making the HIV epidemic difficult to control. Nurses who provide care in the public sector in South Africa (SA) are the frontline providers of HIV care and experience extremely high levels of stress as a consequence of limited resources, hindering their ability to provide effective care, a situation that has been exacerbated by the ongoing COVID-19 pandemic. This study will tailor an established stress management and resilience-enhancing intervention to meet the needs and improve the well-being of nurses providing HIV care in the public sector in SA, and then evaluate the feasibility and acceptability of our program, with the goal of developing a scalable intervention that could be used across similar settings worldwide to improve the treatment and care of PLWH.

CRITIQUE 1

Significance: 4
Investigator(s): 2
Innovation: 2
Approach: 4
Environment: 1

Overall Impact: This is a revised R34 to adapt an existing mind-body skills based intervention (the Relaxation Response Resiliency Program) to improve resiliency against occupational stress among nurses who provide HIV care and treatment in South Africa and ultimately improve patient outcomes. The premise is that provider stress affects quality of care and subsequently affects patient outcomes. The proposed research is responsive to NIH and OAR priorities, and especially timely given the extreme demands on health care providers during what is now a "third wave" of COVID-19 in South Africa. The team is highly qualified with experience in HIV counseling, resiliency interventions, nursing, health systems in SA, and relevant methodologic experience. The team has a history of working together, and has experience adapting the intervention for use in other populations. The proposed work is innovative given the dearth of evidence regarding interventions for stress reduction that target health providers in sub-Saharan Africa. The environment is strong. The revision is responsive to the prior review. There are minor though modifiable weaknesses in the approach. However, these concerns are minor and do not diminish enthusiasm for this strong proposal with strong potential for a high level of impact.

1. Significance:

Strengths

- The proposed intervention focuses on addressing and reducing work-related stress amongst nurses in the public sector in South Africa. The extent of the problem is well-documented & has been shown to be associated with adverse patient outcomes including decreased patient

satisfaction, increased post-hospitalization recovery time, reduced ART adherence, testing, uptake and PMTCT care.

- Addressing the needs of nurses is important given their contribution to care at all points along the HIV care cascade
- Highly responsive to PA-20-141 and OAR priorities

Weaknesses

- Patients typically interact with multiple personnel during clinic visits – any one of those interactions has the potential to be perceived/experienced negatively. While it is understood that nurses are responsible for the bulk of direct care, for real systemic change to be occur and to be sustained, shouldn't all personnel at participating clinics be trained?

2. Investigator(s):

Strengths

- The team is well-qualified with an established record of prior work together
- The team has experience adapting and testing the 3RP intervention with other populations

Weaknesses

- None noted.

3. Innovation:

Strengths

- Would be amongst the first interventions to intervene at the provider level in SA
- Would be amongst the first proposals funded by NH to address nurse stress
- Would be the first to link clinician-level delivery of the 3RP program to patient outcomes amongst people living with HIV

Weaknesses

- None noted.

4. Approach:

Strengths

- Approach is appropriate / relevant to the context – e.g., will explore delivery by non-mental health clinician and explore novel and flexible means of delivery using virtual platforms and other technology
- Will use ORBIT model to guide adaptation / design
- Iterative process for eliciting feedback on 3RP adaptation at MGH and consultation with the DOH are strengths
- Clinic level randomization is appropriate
- Clearly defined and justified cut points for feasibility and acceptability
- High level of attention to monitoring and fidelity

Weaknesses

- Self-administration of survey with questions related to patient experiences with HIV is not optimal. Patients with lower levels of literacy are less likely to opt in as participants yet are the patients who are most likely to have negative experiences with health providers. The approach will also make it difficult if not impossible to determine the denominator of patients who are eligible to participate.

5. Environment:

Strengths

- Excellent and supportive environments to support the proposed work

Weaknesses

- None noted

Study Timeline:

Strengths

- The overall phased plan is thorough, detailed and appropriate.

Weaknesses

- None noted

Protections for Human Subjects:

Acceptable Risks and/or Adequate Protections

- What steps will be taken to ensure that nurses will not be coerced to participate by their supervisors?

Data and Safety Monitoring Plan (Applicable for Clinical Trials Only):

Acceptable

Inclusion Plans:

- Sex/Gender: Distribution justified scientifically
- Race/Ethnicity: Distribution justified scientifically
- For NIH-Defined Phase III trials, Plans for valid design and analysis: Not applicable
- Inclusion/Exclusion Based on Age: Distribution justified scientifically

Vertebrate Animals:

Not Applicable (No Vertebrate Animals)

Biohazards:

Not Applicable (No Biohazards)

Resubmission:

- This revision is responsive to the prior review.

Resource Sharing Plans:

Not Applicable (No Relevant Resources)

Budget and Period of Support:

Recommend as Requested

CRITIQUE 2

Significance: 6
Investigator(s): 3
Innovation: 2
Approach: 6
Environment: 3

Overall Impact: The revised R34 application seeks to adapt an intervention to improve resiliency to buffer occupational stress among HIV nurses in South Africa with a long-term goal of determining if this improves HIV care outcomes among patients. The issue of stress and burnout among HIV nurses is a significant and urgent one, and the investigators thoughtfully describe the hypothesized mechanism by which burnout negatively impacts HIV outcomes. The approach is novel, team strong, and the environment is supportive the study. The approach also contains many rigorous features including using a well-established intervention, clinic level randomization, pragmatic and flexible approach to intervention delivery, and *a priori* feasibility and acceptability metrics. Further, the team has been mostly responsive to previous reviews. While the application is responsive and well-constructed, there are several issues that lead this reviewer to question whether the study will be able to have a sustained impact on the field. Primarily, the study's singular focus on building stress and resilience of the *individual* nurses at the expense of widening the intervention targets to include the more powerful structural contributors to stress and burnout (detail below). Additionally, the team only includes nurses as interventionalists, not as scientific investigators, which may lead to an intervention that is not wholly resonant with the many HIV nurses in South Africa. Finally, it is still unclear how the anonymous patient surveys will lead to valid data on intervention's impact on patient outcomes. Overall, these are significant issues that diminish the study's potential impact.

1. Significance:

Strengths

- The proposal's focus on increasing resilience in highly stressed providers in South Africa is both novel and significant and may lead to discoveries that will dramatically improve clinical care and public policy
- The study focus is consistent with the PAR
- The detailed description of how nurse burnout influences HIV outcomes among patients supports the study's scientific premise
- Expansion of the 3RP to both PWH outcomes and LMICs will help to advance our understanding of how to adapt this intervention into new settings

Weaknesses

- Stress and burnout among healthcare workers, and nurses in particular, are significant and urgent problems that impact health care outcomes; however it is unclear if the structural causes of stress and burnout can be effectively addressed by an intervention that targets the individual's resilience. There is a risk that the data could result in nurses being blamed for not being resilient enough leading to poor patient outcomes, while ignoring the significant structural issues (e.g., workforce shortages, poor pay, little respect pervasive discrimination, and few necessary resources (including PPE)) that underlie the stress and burnout. The singular focus on stress and resilience of the *individual nurses*, is unlikely to lead to an intervention that will have a sustained impact on the field.

2. Investigator(s):

Strengths

- Team has expertise in HIV and women (Psaros), resiliency interventions (Park), nursing in South Africa (Ralfe), health systems research in South Africa (Smit), qualitative and mixed methods (Traeger), and statistics (Vangel)
- The addition of Dr. Park and the expansion of the expertise of Drs Smith and Traeger are responsive the previous review and should help the team develop interventions for nurses in South Africa

Weaknesses

- None noted

3. Innovation:

Strengths

- Exploring the potential use of remote digital technology for 3RP is a novel method for an established in person intervention
- Similarly, adapting 3Rp to be delivered by nurses instead of mental health providers is a novel approach that could enhance the scalability of the intervention (should it be found to work)

Weaknesses

- None noted

4. Approach:

Strengths

- The 3RP conceptual model and intervention is supported by a strong body of literature, including among oncology nurses in the US
- Clinic level randomization and matching scheme is a rigorous feature of the approach
- Flexible approach to intervention delivery will increase the likelihood that nurses will be able to complete the intervention
- The inclusion of *a priori* thresholds for acceptability and feasibility is a strength

Weaknesses

- Will the team that is adapting the intervention meaningfully include nurses who are currently practicing in HIV care in South Africa?
- Questions about the appropriateness of patient-level surveys on HIV outcomes remain. Specifically, if the surveys are not matched to the clinic nurses' completion of the intervention, how valid will the results of the patient surveys be? There is a concern that by simply receiving a lot of completed surveys, the method will be deemed "feasible" and "acceptable". There is no discussion of the validity of this approach.

5. Environment:

Strengths

- Mass General and the MaTCH research contain a number of resources that should support the team in completing the project's aims

Weaknesses

- Exactly how the listed resources in these settings will support the study activities is not described

Study Timeline:

Strengths

- Timeline is consistent with the R34 mechanism

Weaknesses

- There is limited detail on the specific activities and milestones in the timeline
- It is likely IRB approval will need to be obtained prior to the first month of funding

Protections for Human Subjects:

Unacceptable Risks and/or Inadequate Protections

- How will the team ensure the nurses are not coerced into participating by their supervisors? Especially if the clinic, and not the individual nurses, will receive a token of appreciation.

Data and Safety Monitoring Plan (Applicable for Clinical Trials Only):

Acceptable

- Addition of the DSMB enhances data safety and is responsive to the prior review

Inclusion Plans:

- Sex/Gender: Distribution justified scientifically
- Race/Ethnicity: Distribution justified scientifically
- For NIH-Defined Phase III trials, Plans for valid design and analysis: Not applicable
- Inclusion/Exclusion Based on Age: Distribution justified scientifically
- The sex/gender, race and age criteria are all scientifically justified by the distribution of nurses in South Africa

Vertebrate Animals:
Not Applicable (No Vertebrate Animals)

Biohazards:
Not Applicable (No Biohazards)

Resubmission:

- The applicants made a number of pragmatic and thoughtful revisions in this resubmission, in response to the prior critique including increasing the sample size, adding team members and deleting references to anticipated effect sizes.

Applications from Foreign Organizations:
Not Applicable (No Foreign Organizations)

- The performance site in South Africa is justified but not necessary since the contact site is a domestic organization

Resource Sharing Plans:
Acceptable

Budget and Period of Support:
Recommend as Requested

CRITIQUE 3

Significance: 2
Investigator(s): 2
Innovation: 2
Approach: 3
Environment: 1

Overall Impact: The investigators propose to adapt and pilot test a stress reduction and resilience intervention (3RP) for South African nurses providing HIV care in the public sector clinics in hopes of improving patients' care provision and therefore engagement in the HIV care cascade. The application is timely, particularly given that there are no interventions for stress reduction in the workforce and SA is entering the 3rd wave of the Covid pandemic with limited vaccine supply and a health care workforce that has spent almost 18 months dealing with the pandemic already. The proposal is well written; the investigators are highly qualified; and the methods are appropriate. There were some minor, addressable concerns, including inadequate explanation of the processes the investigators will use to determine patient outcomes for future trials, the lack of a primary outcome of provider stress, and clarity around how many clinics will be included in this research. These concerns do not diminish the fact that this is a strong proposal with high potential for impact.

1. Significance:

Strengths

- Nurses in SA experience high levels of burnout, which can have deleterious results for the provider, the health care team and clinic context and the patients in care. This feeds the shortage of health care workers.
- There are no known interventions to reduce stress and build resiliency (or reduce burnout) for the health care workforce that have been utilized in SSA, but there is precedent for stress reduction and resiliency interventions in other contexts.
- KwaZulu-Natal (KZN) has the highest HIV prevalence in the country (and one of the highest in the world) -- 40.8% of adults over 15 are living with HIV.
- Burnout and work-related stress discourage more people from entering the workforce (in SA there is a shortage of nurses).
- Addressing the context of health care and the providers represents a structural approach and has potential for broader impact.

Weaknesses

- Are there precedents for this kind of intervention in other parts of SSA or other LMICs? Examples are provided from the US, but it's unclear if there is research from any lower income contexts and countries that could provide important insights.

2. Investigator(s):

Strengths

- PI Dr. Psaros is well qualified - an Associate Professor of Psychology in the Department of Psychiatry at Harvard Medical School and the Associate Director of the Behavioral Medicine Program at Mass General.
- Strong team of Co-Is with relevant expertise and experience.

Weaknesses

- Unclear if there will be include input from local nurses and / or the South African Clinician's Society.

3. Innovation:

Strengths

- Targeting the health care workforce can impact numerous patients with potential for broader impact.
- Stress and burnout are extremely common in the SA workforce, but there have been no interventions to support them.
- Exploring delivery by a non-mental health specialist (e.g., a nurse) is important – given the lack of availability of mental health clinicians in SA and the peer-based experience needed to truly understand the context of stress.

Weaknesses

- Linking provider stress management to improved patient outcomes is listed as an innovation, but it is unclear how investigators will track patient outcomes and link them to the intervention.

The authors note that this is being explored, but with ample experience in South Africa, there should be some potential mechanisms presented – e.g. through Tier.net indicators?

4. Approach:

Strengths

- Clear and appropriate measures of feasibility and acceptability as well as patient satisfaction through patient exit interviews.
- Following the ORBIT model to adapt and refine the 3RP intervention – an evidence-based intervention developed by the Co-I team in the US.
- Strong theoretical model and foundation of the 3RP intervention.
- Refinements of the 3RP conducted in two stages, first through formative research and then through a small proof-of-concept study.
- Appropriate control condition provided. In addition, control participants have the option of receiving the intervention following the RCT.

Weaknesses

- It is understandable that the investigators will not assess preliminary efficacy regarding impacts on patient outcomes, however, it is not clear why the investigators do not intend to assess changes in perceived stress and resilience among providers as a primary outcome. Evidence that this intervention works in terms of stress reduction in SA would be needed for a future trial, particularly if the final outcome of interest (e.g. patient outcomes) is one more step removed.
- Unclear how many clinics will be included among the approximately 60 clinics? and how would the investigators know the sample of selected nurses is representative? Also not stated how many clinics will be included in recruitment of patients for the anonymous patient survey.
- The investigators will collect data on patient's treatment history to assess which patient outcomes could be utilized in the future trial (R01). It's unclear if this data is coming from the anonymous patient surveys or from clinic records and how many clinics will they elicit this data from? If from clinic records, will they request this data from the national reporting system or extract this from charts? The description of this exploratory aspect of the research is quite vague.

5. Environment:

Strengths

- Both partnering institutions have strong resources to support the planned research.
- Support from Department of Health

Weaknesses

- None noted

Study Timeline:

Strengths

- Reasonable time allocated to Aim 1 (refining the intervention).

Weaknesses

- Little time for RCT data analysis

Protections for Human Subjects:
Acceptable Risks and/or Adequate Protections
Data and Safety Monitoring Plan (Applicable for Clinical Trials Only):
Acceptable

Inclusion Plans:
- Sex/Gender: Distribution justified scientifically
- Race/Ethnicity: Distribution justified scientifically
- For NIH-Defined Phase III trials, Plans for valid design and analysis: Not applicable
- Inclusion/Exclusion Based on Age: Distribution justified scientifically
- Sex/Gender, Race/Ethnicity, Age inclusion all justified.

Vertebrate Animals:
Not Applicable (No Vertebrate Animals)

Biohazards:
Not Applicable (No Biohazards)

Resubmission:
- Responsive to prior reviews

Resource Sharing Plans:
Acceptable

Budget and Period of Support:
Recommend as Requested
Recommended budget modifications or possible overlap identified:
- Time for Project Director in US is limited (only 0.6 calendar months in years 1 and 3 will make it difficult to cover all of the responsibilities listed).

THE FOLLOWING SECTIONS WERE PREPARED BY THE SCIENTIFIC REVIEW OFFICER TO SUMMARIZE THE OUTCOME OF DISCUSSIONS OF THE REVIEW COMMITTEE, OR REVIEWERS' WRITTEN CRITIQUES, ON THE FOLLOWING ISSUES:

PROTECTION OF HUMAN SUBJECTS: ACCEPTABLE

INCLUSION OF WOMEN PLAN: ACCEPTABLE

INCLUSION OF MINORITIES PLAN: ACCEPTABLE

INCLUSION ACROSS THE LIFESPAN: ACCEPTABLE

COMMITTEE BUDGET RECOMMENDATIONS: The budget was recommended as requested.

Footnotes for 1 R34 MH126753-01A1; PI Name: Psaros, Christina

+ Derived from the range of percentile values calculated for the study section that reviewed this application.

NIH has modified its policy regarding the receipt of resubmissions (amended applications).See Guide Notice NOT-OD-18-197 at https://grants.nih.gov/grants/guide/notice-files/NOT-OD-18-197.html. The impact/priority score is calculated after discussion of an application by averaging the overall scores (1-9) given by all voting reviewers on the committee and multiplying by 10. The criterion scores are submitted prior to the meeting by the individual reviewers assigned to an application, and are not discussed specifically at the review meeting or calculated into the overall impact score. Some applications also receive a percentile ranking. For details on the review process, see http://grants.nih.gov/grants/peer_review_process.htm#scoring.

MEETING ROSTER

HIV/AIDS Intra- and Inter-personal Determinants and Behavioral Interventions Study Section
Risk, Prevention and Health Behavior Integrated Review Group
CENTER FOR SCIENTIFIC REVIEW
HIBI
07/12/2021 - 07/13/2021

Notice of NIH Policy to All Applicants: Meeting rosters are provided for information purposes only. Applicant investigators and institutional officials must not communicate directly with study section members about an application before or after the review. Failure to observe this policy will create a serious breach of integrity in the peer review process, and may lead to actions outlined in NOT-OD-14-073 at https://grants.nih.gov/grants/guide/notice-files/NOT-OD-14-073.html, NOT-OD-15-106 at https://grants.nih.gov/grants/guide/notice-files/NOT-OD-15-106.html, and NOT-OD-18-115 at https://grants.nih.gov/grants/guide/notice-files/NOT-OD-18-115.html, including removal of the application from immediate review.

CHAIRPERSON(S)

KIPKE, MICHELE D, PHD
PROFESSOR
DEPARTMENTS OF PEDIATRICS
AND PREVENTIVE MEDICINE
KECK SCHOOL OF MEDICINE
UNIVERSITY OF SOUTHERN CALIFORNIA
LOS ANGELES, CA 90028

MEMBERS

BUTLER, LISA MICHELLE, PHD
ASSOCIATE RESEARCH PROFESSOR
INSTITUTE FOR COLLABORATION ON HEALTH,
INTERVENTION, AND POLICY
UNIVERSITY OF CONNECTICUT
STORRS, CT 06269

COMULADA, WARREN SCOTT, DRPH
ASSOCIATE PROFESSOR
DEPARTMENT OF PSYCHIATRY
AND BIOBEHAVIORAL SCIENCES
SCHOOL OF PUBLIC HEALTH
UNIVERSITY OF CALIFORNIA, LOS ANGELES
LOS ANGELES, CA 90024

DODGE, BRIAN MARK, PHD
PROFESSOR
DEPARTMENT OF APPLIED HEALTH SCIENCE
INDIANA UNIVERSITY SCHOOL OF PUBLIC HEALTH
BLOOMINGTON, IN 47405

GRAHAM, SUSAN MARIE, MD, PHD
PROFESSOR
DIVISION OF ALLERGY AND INFECTIOUS DISEASES
DEPARTMENTS OF MEDICINE AND GLOBAL HEALTH
SCHOOL OF MEDICINE
UNIVERSITY OF WASHINGTON
SEATTLE, WA 98104

GROV, CHRISTIAN, PHD
PROFESSOR AND CHAIR
DEPARTMENT OF COMMUNITY HEALTH
AND SOCIAL SCIENCES
SCHOOL OF PUBLIC HEALTH AND HEALTH POLICY
CITY UNIVERSITY OF NEW YORK
NEW YORK, NY 10027

HANSEN, NATHAN B, PHD
DEPARTMENT HEAD AND PROFESSOR
DEPARTMENT OF HEALTH PROMOTION AND BEHAVIOR
COLLEGE OF PUBLIC HEALTH
UNIVERSITY OF GEORGIA
ATHENS, GA 30602

HORVATH, KEITH JOSEPH, PHD
ASSOCIATE PROFESSOR
DEPARTMENT OF CLINICAL PSYCHOLOGY
SAN DIEGO STATE UNIVERSITY
SAN DIEGO, CA 92120

IWELUNMOR, JULIET, PHD
ASSOCIATE PROFESSOR
DEPARTMENT OF BEHAVIORAL SCIENCE AND
HEALTH EDUCATION
COLLEGE FOR PUBLIC HEALTH AND SOCIAL JUSTICE
ST. LOUIS UNIVERSITY
ST. LOUIS, MO 63104

JOHNSON, DAWN M, PHD *
ASSOCIATE PROFESSOR
DEPARTMENT OF PSYCHOLOGY
UNIVERSITY OF AKRON
AKRON, OH 44325

LIPPMAN, SHERI ANN, PHD *
ASSOCIATE PROFESSOR
CENTER FOR AIDS PREVENTION STUDIES
UNIVERSITY OF CALIFORNIA, SAN FRANCISCO
SAN FRANCISCO, CA 94143

LOVEJOY, TRAVIS IAN, PHD
ASSOCIATE PROFESSOR
DEPARTMENT OF PSYCHIATRY
SCHOOL OF MEDICINE
OREGON HEALTH AND SCIENCE UNIVERSITY
PORTLAND, OR 97239

MACDONELL, KAREN KOLMODIN, PHD *
ASSOCIATE PROFESSOR
DEPARTMENT OF FAMILY MEDICINE
AND PUBLIC HEALTH SCIENCES
SCHOOL OF MEDICINE
WAYNE STATE UNIVERSITY
DETROIT, MI 48202

MUESSIG, KATHRYN E, PHD *
ASSISTANT PROFESSOR
DEPARTMENT OF HEALTH BEHAVIOR
GILLINGS SCHOOL OF GLOBAL PUBLIC HEALTH
UNIVERSITY OF NORTH CAROLINA AT CHAPEL HILL
CHAPEL HILL, NC 27599

OWCZARZAK, JILL, PHD *
ASSOCIATE PROFESSOR
DEPARTMENT OF HEALTH, BEHAVIOR AND SOCIETY
BLOOMBERG SCHOOL OF PUBLIC HEALTH
JOHNS HOPKINS UNIVERSITY
BALTIMORE, MD 21205

PATEL, VIRAJ V, MD, MPH *
ASSOCIATE PROFESSOR
DEPARTMENT OF MEDICINE
ALBERT EINSTEIN COLLEGE OF MEDICINE
BRONX, NY 10461

RAEL, CHRISTINE TAGLIAFERRI, PHD *
ASSISTANT PROFESSOR
COLLEGE OF NURSING
UNIVERSITY OF COLORADO ANSCHUTZ MEDICAL CAMPUS
AURORA, CO 80045

RAMSEY, SUSAN E, PHD
ASSOCIATE PROFESSOR
DIVISION OF GENERAL INTERNAL MEDICINE
RHODE ISLAND HOSPITAL
BROWN UNIVERSITY
PROVIDENCE, RI 02903

RODRIGUEZ-DIAZ, CARLOS EMANUEL, PHD *
ASSOCIATE PROFESSOR
DEPARTMENT OF PREVENTION AND COMMUNITY HEALTH
MILKEN INSTITUTE SCHOOL OF PUBLIC HEALTH
THE GEORGE WASHINGTON UNIVERSITY
WASHINGTON, DC 20052

SAFREN, STEVEN A, PHD
PROFESSOR
DEPARTMENT OF PSYCHOLOGY
COLLEGE OF ARTS AND SCIENCES
UNIVERSITY OF MIAMI
CORAL GABLES, FL 33124

SICONOLFI, DANIEL, MPH, PHD *
BEHAVIORAL SCIENTIST
RAND CORPORATION
PITTSBURGH, PA 15213

SSEWAMALA, FRED M, PHD
PROFESSOR
INSTITUTE FOR PUBLIC HEALTH
BROWN SCHOOL
WASHINGTON UNIVERSITY
ST. LOUIS, MO 63130

STOCKMAN, JAMILA KINSHASA, PHD
PROFESSOR
DIVISION OF GLOBAL PUBLIC HEALTH
DEPARTMENT OF MEDICINE
SCHOOL OF MEDICINE
UNIVERSITY OF CALIFORNIA, SAN DIEGO
LA JOLLA, CA 92093

SULLIVAN, PATRICK SEAN, PHD
PROFESSOR
DEPARTMENT OF EPIDEMIOLOGY
ROLLINS SCHOOL OF PUBLIC HEALTH
EMORY UNIVERSITY
ATLANTA, GA 30322

TANNER, AMANDA E, MPH, PHD *
ASSOCIATE PROFESSOR
DEPARTMENT OF PUBLIC HEALTH EDUCATION
SCHOOL OF HEALTH AND HUMAN SCIENCES
UNIVERSITY OF NORTH CAROLINA GREENSBORO
GREENSBORO, NC 27402

THAMES, APRIL D, PHD
ASSOCIATE PROFESSOR
DEPARTMENT OF PSYCHOLOGY
UNIVERSITY OF SOUTHERN CALIFORNIA
LOS ANGELES, CA 90089

THIELMAN, NATHAN M, MD, MPH *
PROFESSOR
DEPARTMENT OF MEDICINE
DUKE UNIVERSITY SCHOOL OF MEDICINE
DURHAM, NC 27710

WEBEL, ALLISON R, PHD
PROFESSOR
SCHOOL OF NURSING
UNIVERSITY OF WASHINGTON
SEATTLE, WA 98195

WILSON, TRACEY ELIZABETH, PHD *
PROFESSOR
DEPARTMENT OF COMMUNITY HEALTH SCIENCES
SCHOOL OF PUBLIC HEALTH
DOWNSTATE MEDICAL CENTER
THE STATE UNIVERSITY OF NEW YORK
BROOKLYN, NY 11203

WILTON, LEO, PHD, MPH
PROFESSOR
DEPARTMENT OF HUMAN DEVELOPMENT
COLLEGE OF COMMUNITY AND PUBLIC AFFAIRS
BINGHAMTON UNIVERSITY
BINGHAMTON, NY 13902

WINDSOR, LILIANE CAMBRAIA, PHD
ASSOCIATE PROFESSOR
SCHOOL OF SOCIAL WORK
THE UNIVERSITY OF ILLINOIS AT URBANA-CHAMPAIGN
URBANA, IL 61801

YBARRA, MICHELE L., PHD, MPH *
CEO AND RESEARCH DIRECTOR
CENTER FOR INNOVATIVE PUBLIC HEALTH RESEARCH
SAN CLEMENTE, CA 92672

SCIENTIFIC REVIEW OFFICER

RUBERT, MARK P, PHD
SCIENTIFIC REVIEW OFFICER
CENTER FOR SCIENTIFIC REVIEW
NATIONAL INSTITUTES OF HEALTH
BETHESDA, MD 20892

EXTRAMURAL SUPPORT ASSISTANT

CAMBRELEN, AMY ANGELA
EXTRAMURAL SUPPORT ASSISTANT
CENTER FOR SCIENTIFIC REVIEW
NATIONAL INSTITUTE OF HEALTH
BETHESDA, MD 20892

* Temporary Member. For grant applications, temporary members may participate in the entire meeting or may review only selected applications as needed.

Consultants are required to absent themselves from the room during the review of any application if their presence would constitute or appear to constitute a conflict of interest.
